# Comparative genomics reveals bamboo feeding adaptability in the giant panda (*Ailuropoda melanoleuca*)

**DOI:** 10.3897/zookeys.923.39665

**Published:** 2020-04-01

**Authors:** Xin He, Walter H. Hsu, Rong Hou, Ying Yao, Qin Xu, Dandan Jiang, Longqiong Wang, Hairui Wang

**Affiliations:** 1 Chengdu Research Base of Giant Panda Breeding, Chengdu, 610081, China; 2 Sichuan Key Laboratory of Conservation Biology for Endangered Wildlife, Chengdu, 610081, China; 3 Sichuan Academy of Giant Panda, Chengdu, 610081, China; 4 Department of Biomedical Sciences, Iowa State University, Ames 50011, IA, USA

**Keywords:** adaptation, bamboo diet, dietary transition, digestion, feeding habits

## Abstract

The giant panda (*Ailuropoda melanoleuca*) is one of the world’s most endangered mammals and remains threatened as a result of intense environmental and anthropogenic pressure. The transformation and specialization of the giant panda’s diet into a herbivorous diet have resulted in unique adaptabilities in many aspects of their biology, physiology and behavior. However, little is known about their adaptability at the molecular level. Through comparative analysis of the giant panda’s genome with those of nine other mammalian species, we found some genetic characteristics of the giant panda that can be associated with adaptive changes for effective digestion of plant material. We also found that giant pandas have similar genetic characteristics to carnivores in terms of olfactory perception but have similar genetic characteristics to herbivores in terms of immunity and hydrolytic enzyme activity. Through the analysis of gene family expansion, 3752 gene families were found, which were enriched in functions such as digestion. A total of 93 genes under positive selection were screened out and gene enrichment identified these genes for the following processes: negative regulation of cellular metabolic process, negative regulation of nitrogen compound metabolic process, negative regulation of macromolecule metabolic process and negative regulation of metabolic process. Combined with the KEGG pathway, it was found that genes such as CREB3L1, CYP450 2S1, HSD11B2, LRPAP1 play a key role in digestion. These genes may have played a key role in the pandas’ adaptation to its bamboo diet.

## Introduction

Diet may be the most important selective force in animal evolution (Kim et al. 2016). In this regard, the giant panda (*Ailuropoda melanoleuca*) is interesting because it has not only undergone a dietary transition but has also developed an obligate bamboo diet (O’Brien 1987; Wei et al. 2015a). The fossil record suggests that giant pandas started to consume bamboo in the late Pliocene or early Pleistocene (Jin et al. 2007). Giant pandas belong to the order Carnivora and have a digestive tract typical of the carnivorous members of the group, but feed exclusively on low nutritious and low-calorific content bamboo (Wei et al. 2012). Although giant pandas have been found to retain the ability to eat meat both in the wild and in captivity, 99% of their diet consists of bamboo (Wei et al. 2015a).

Bamboo is a low nutrition/energy food comprising of 70–80% cellulose, hemicellulose, and lignin and 20–30% protein, soluble carbohydrate, and fat (Wu et al. 2017). Meanwhile, giant pandas have a very low digestive capacity for bamboo: 75–90% of the protein, only 27% of the hemicellulose, and 8% of the cellulose is utilized (Zhang et al. 2014). Despite this, they have survived on a bamboo diet probably for more than 2 million years (Myr). The monotonous diet has probably resulted in a number of morphological, ecological and genetic adaptations (Wei et al. 2015b). Giant pandas have a pseudothumb on the forepaw, and have evolved a strong mandible and flattened teeth to help them chew and eat bamboo (Endo et al. 1999; Zhao et al. 2010). Giant pandas have acquired a suite of optimal foraging, habitat use, and activity rhythm strategies in adapting to their low energy diet (O’Brien 1987; Zhang et al. 2014; Nie et al. 2015b). The study of the giant panda genome reveals that the umami receptor TAS1R1 gene has become a pseudogene due to a 2 bp insertion in exon 3 and a 6 bp deletion in exon 6 (Li et al. 2010). The umami receptor senses components of meat and other protein-rich foods. Therefore, the loss of function of the TAS1R1 gene may have contributed to the panda’s dietary switch (Zhao et al. 2010). In addition, little is known about the molecular mechanisms by which giant pandas adapt to bamboo diets. Some studies have shown that giant panda’s digestion of bamboo mainly depends on its gut microbiota (Li et al. 2010; Wei et al. 2015b; Guo et al. 2018; Zhang et al. 2018). It is unknown as to what caused the adaptive changes in giant pandas.

The genome sequences of wild animal species are rapidly being accumulated, providing rich resources for the study of adaptation, trait evolution, species divergence, and population structure analyses (Li et al. 2010; Kim et al. 2016). Here, we investigated the giant panda’s adaptation to a bamboo diet using comparative genomics among mammalian species with different feeding habits. In the present study, we detected putative molecular adaptation mechanisms of giant pandas to the bamboo diet, providing new insights into the research and protection of giant pandas.

## Materials and methods

### Abbreviations

**Myr**: million years; **BLAST**: basic local alignment search tool; **GO**: gene ontology; **KEGG**: Kyoto Encyclopedia of Genes and Genomes; **NCBI**: National Center for Biotechnology Information; **dN/dS**: non-synonymous/synonymous rate ratio; **ML**: maximum likelihood; **CREB3L1**: cAMP-responsive element binding protein 3 like 1; **CYP450 2S1**: cytochrome P450, family 2, subfamily s, polypeptide 1; **HSD11B2**: corticosteroid 11-beta-dehydrogenase isozyme 2; **LRPAP1**: low density lipoprotein receptor related protein associated protein 1

### Genome data query

We performed comparative genomic analysis of 10 mammalian species with different diets including the giant panda (*Ailuropoda melanoleuca*); four species of mammals with carnivorous diets: wolf (*Canis lupus familiaris*), tiger (*Panthera tiger*) polar bear (*Ursus maritimus*) and sperm whale (*Physeter catodon*)); two species with herbivorous diets: white rhinoceros (*Ceratotherium simum simum*) and gorilla (*Gorilla gorilla*); and three species of omnivores: macaque (*Macaca mulatta*), human (*Homo sapiens*) and mouse (*Mus musculus*). For the present study, we downloaded the genome sequences, protein sequences and annotation files of the 10 species from the NCBI website (2018/3/7) (Table 1).

**Table 1.** Data sources of comparative genomics of the giant panda.

Species	Download Website
*Ailuropoda melanoleuca*	ftp://ftp.ncbi.nlm.nih.gov/genomes/all/GCF/000/004/335/GCF_000004335.2_AilMel_1.0/GCF_000004335.2_AilMel_1.0_genomic.fna.gz
	ftp://ftp.ncbi.nlm.nih.gov/genomes/all/GCF/000/004/335/GCF_000004335.2_AilMel_1.0/GCF_000004335.2_AilMel_1.0_protein.faa.gz
	ftp://ftp.ncbi.nlm.nih.gov/genomes/all/GCF/000/004/335/GCF_000004335.2_AilMel_1.0/GCF_000004335.2_AilMel_1.0_genomic.gff.gz
*Canis lupus familiaris*	ftp://ftp.ncbi.nlm.nih.gov/genomes/all/GCF/000/002/285/GCF_000002285.3_CanFam3.1/GCF_000002285.3_CanFam3.1_genomic.fna.gz
	ftp://ftp.ncbi.nlm.nih.gov/genomes/all/GCF/000/002/285/GCF_000002285.3_CanFam3.1/GCF_000002285.3_CanFam3.1_protein.faa.gz
	ftp://ftp.ncbi.nlm.nih.gov/genomes/all/GCF/000/002/285/GCF_000002285.3_CanFam3.1/GCF_000002285.3_CanFam3.1_genomic.gff.gz
*Ceratotherium simum simum*	ftp://ftp.ncbi.nlm.nih.gov/genomes/all/GCF/000/283/155/GCF_000283155.1_CerSimSim1.0/GCF_000283155.1_CerSimSim1.0_genomic.fna.gz
	ftp://ftp.ncbi.nlm.nih.gov/genomes/all/GCF/000/283/155/GCF_000283155.1_CerSimSim1.0/GCF_000283155.1_CerSimSim1.0_protein.faa.gz
	ftp://ftp.ncbi.nlm.nih.gov/genomes/all/GCF/000/283/155/GCF_000283155.1_CerSimSim1.0/GCF_000283155.1_CerSimSim1.0_genomic.gff.gz
*Gorilla gorilla*	ftp://ftp.ncbi.nlm.nih.gov/genomes/all/GCF/000/151/905/GCF_000151905.2_gorGor4/GCF_000151905.2_gorGor4_genomic.fna.gz
	ftp://ftp.ncbi.nlm.nih.gov/genomes/all/GCF/000/151/905/GCF_000151905.2_gorGor4/GCF_000151905.2_gorGor4_protein.faa.gz
	ftp://ftp.ncbi.nlm.nih.gov/genomes/all/GCF/000/151/905/GCF_000151905.2_gorGor4/GCF_000151905.2_gorGor4_genomic.gff.gz
*Homo sapiens*	ftp://ftp.ncbi.nlm.nih.gov/genomes/all/GCF/000/001/405/GCF_000001405.38_GRCh38.p12/GCF_000001405.38_GRCh38.p12_genomic.fna.gz
	ftp://ftp.ncbi.nlm.nih.gov/genomes/all/GCF/000/001/405/GCF_000001405.38_GRCh38.p12/GCF_000001405.38_GRCh38.p12_protein.faa.gz
	ftp://ftp.ncbi.nlm.nih.gov/genomes/all/GCF/000/001/405/GCF_000001405.38_GRCh38.p12/GCF_000001405.38_GRCh38.p12_genomic.gff.gz
*Macaca mulatta*	ftp://ftp.ncbi.nlm.nih.gov/genomes/all/GCF/000/772/875/GCF_000772875.2_Mmul_8.0.1/GCF_000772875.2_Mmul_8.0.1_genomic.fna.gz
	ftp://ftp.ncbi.nlm.nih.gov/genomes/all/GCF/000/772/875/GCF_000772875.2_Mmul_8.0.1/GCF_000772875.2_Mmul_8.0.1_protein.faa.gz
	ftp://ftp.ncbi.nlm.nih.gov/genomes/all/GCF/000/772/875/GCF_000772875.2_Mmul_8.0.1/GCF_000772875.2_Mmul_8.0.1_genomic.gff.gz
*Mus musculus*	ftp://ftp.ncbi.nlm.nih.gov/genomes/all/GCF/000/001/635/GCF_000001635.26_GRCm38.p6/GCF_000001635.26_GRCm38.p6_genomic.fna.gz
	ftp://ftp.ncbi.nlm.nih.gov/genomes/all/GCF/000/001/635/GCF_000001635.26_GRCm38.p6/GCF_000001635.26_GRCm38.p6_protein.faa.gz
	ftp://ftp.ncbi.nlm.nih.gov/genomes/all/GCF/000/001/635/GCF_000001635.26_GRCm38.p6/GCF_000001635.26_GRCm38.p6_genomic.gff.gz
*Panthera tiger*	ftp://ftp.ncbi.nlm.nih.gov/genomes/all/GCF/000/464/555/GCF_000464555.1_PanTig1.0/GCF_000464555.1_PanTig1.0_genomic.fna.gz
	ftp://ftp.ncbi.nlm.nih.gov/genomes/all/GCF/000/464/555/GCF_000464555.1_PanTig1.0/GCF_000464555.1_PanTig1.0_protein.faa.gz
	ftp://ftp.ncbi.nlm.nih.gov/genomes/all/GCF/000/464/555/GCF_000464555.1_PanTig1.0/GCF_000464555.1_PanTig1.0_genomic.gff.gz
*Physeter catodon*	ftp://ftp.ncbi.nlm.nih.gov/genomes/all/GCF/002/837/175/GCF_002837175.1_ASM283717v1/GCF_002837175.1_ASM283717v1_genomic.fna.gz
	ftp://ftp.ncbi.nlm.nih.gov/genomes/all/GCF/002/837/175/GCF_002837175.1_ASM283717v1/GCF_002837175.1_ASM283717v1_protein.faa.gz
	ftp://ftp.ncbi.nlm.nih.gov/genomes/all/GCF/002/837/175/GCF_002837175.1_ASM283717v1/GCF_002837175.1_ASM283717v1_genomic.gff.gz
*Ursus maritimus*	ftp://ftp.ncbi.nlm.nih.gov/genomes/all/GCF/000/687/225/GCF_000687225.1_UrsMar_1.0/GCF_000687225.1_UrsMar_1.0_genomic.fna.gz
	ftp://ftp.ncbi.nlm.nih.gov/genomes/all/GCF/000/687/225/GCF_000687225.1_UrsMar_1.0/GCF_000687225.1_UrsMar_1.0_protein.faa.gz
	ftp://ftp.ncbi.nlm.nih.gov/genomes/all/GCF/000/687/225/GCF_000687225.1_UrsMar_1.0/GCF_000687225.1_UrsMar_1.0_genomic.gff.gz

### Gene family analysis

OrthoFinder (OrthoFinder-2.2.7) software was used for the gene orthology analysis (Emms and Kelly 2015). We first performed all-blast-all alignment of the protein sequences of all species, and then clustered these proteins according to the alignment results; each of which is a gene family. Based on the results of the gene family analysis, the common and unique gene families shared between the giant panda and the carnivore and herbivore groups were analyzed. A Venn diagram was used to depict the common and unique gene families. These genes were enriched and annotated by Go-TermFinder (Boyle et al. 2004) and KEGG pathways (Kanehisa 2002; Kanehisa et al. 2004) to analyze the differences in gene function.

### Comparative evolution analysis

The single-copy gene protein sequences were compared using the MAFFT (v7.158b (2014/06/27)) software (Katoh et al. 2005); the regions with poor comparison quality were removed using the trimAl (v1.4.rev22) software (Capella-Gutiérrez et al. 2009), and the parameter was set to automated1. Subsequently, 1000 times of the bootstrap test were performed using the PROTGAMMAJTT model, RAxML (v8.2.12) software (Stamatakis 2014) to construct a ML evolutionary tree. Based on the results of the single-copy gene alignment, the MCMCtree program with the Paml software (Yang 1997) was used to estimate the divergence time between species. The fossil time was calibrated as follows: a) divergence time between dogs and cats was > 43 Myr and < 65 Myr (Wesley‐Hunt and Flynn 2005; Eizirik et al. 2010; Meredith et al. 2011); b) primates and rodents diverged > 65 Myr (Wesley‐Hunt and Flynn 2005); c) Canidae and Ursidae diverged >37 Myr (Wesley‐Hunt and Flynn 2005). By using different random numbers and running the program twice, we found that the correlation between the two estimates was 0.999, indicating that the time accuracy of this estimation was very high. The Cafe software (De Bie et al. 2006) was used to estimate the expansion and contraction of gene families, and the Go-TermFinder (Boyle et al. 2004) was used to analyze the GO enrichment annotation of these genes and the KEGG pathway (Kanehisa et al. 2004) enrichment.

### Positive selection gene

The giant panda gene was chosen as the foreground and the genes of the remaining species as the background. The positive selection analysis was used to determine whether there was a significant difference between the non-synonymous replacement rate and the proportion of synonymous replacement rates (dN/dS) between foreground and background branches. The null hypothesis parameters were: model = 2, NSsites = 2, fix_omega = 1, omega = 1. Alternative hypothesis parameters were: model = 2, NSsites = 2, fix_omega = 0. Chi programs using Paml software (Yang 1997) were utilized to compare LRT differences between the two models. The QVALUE of R package (Halekoh et al. 2006) was used for multiple inspections and corrections. Identification of genes that were under positive selection in the giant panda branch ensued. The GO enrichment annotation analysis was performed on these genes using Go-TermFinder (Boyle et al. 2004), and the KEGG pathway (Kanehisa et al. 2004) enrichment analysis was also performed.

## Results

### Gene family

**Figure 1.**
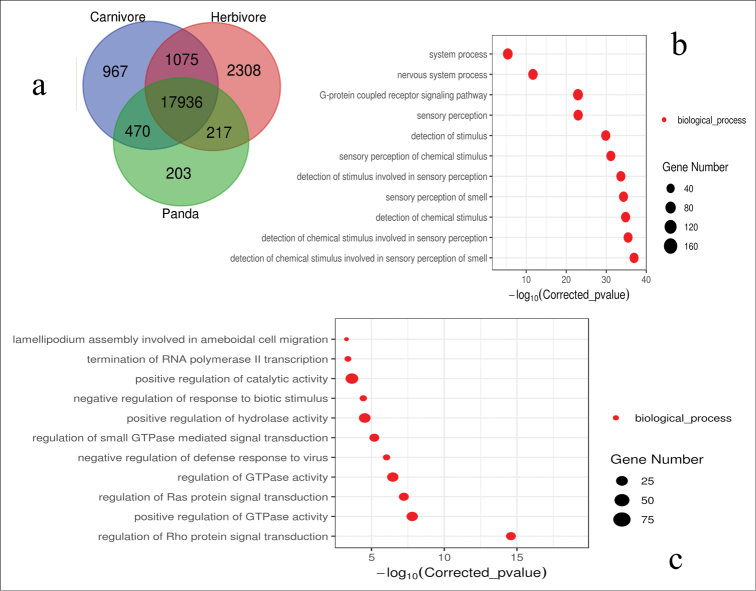
The enrichment analysis of shared genes between the giant panda and mammalian species with different feeding habits. Giant pandas have the characteristics of both carnivores and herbivores. Studies show that it is close to carnivores in perception and close to herbivores in physiological functions. The abscissa is the pair value of the corrected p value, and the corrected p < 0.05 is taken as the threshold value. **a** shared genes between the giant panda and other mammalian species with different feeding habits **b** gene enrichment analysis of the giant panda and carnivores **c** gene enrichment analysis of the giant panda and herbivores.

A total of 517,058 protein-coding genes from 10 species were used for the gene family analysis; 481,081 genes were identified in 24,788 gene families, including 911 single-copy true orthologous genes across all 10 species (Table 2). There were 470 shared gene families in giant pandas and carnivores, and these gene families are enriched in the sense of smell and chemical stimulus detection awareness, etc. At the same time, there were 217 gene families shared with herbivores, which are enriched in virus defense mechanisms, positive regulation of hydrolase activity, negative regulation of biological stimulation and positive regulation of catalytic activity (Fig. 1).

**Table 2.** Statistical results of gene families.

Property	Value
Number of genes	517,058
Number of genes in orthogroups	481,081
Number of unassigned genes	35,977
Percentage of genes in orthogroups	93.00%
Percentage of unassigned genes	7.00%
Number of orthogroups	24,788
Number of species-specific orthogroups	169
Number of genes in species-specific orthogroups	1108
Percentage of genes in species-specific orthogroups	0.20%
Mean orthogroup size	19.4
Median orthogroup size	15
Number of orthogroups with all species present	14,680
Number of single-copy orthogroups	911

### Phylogenetic analysis

**Figure 2.**
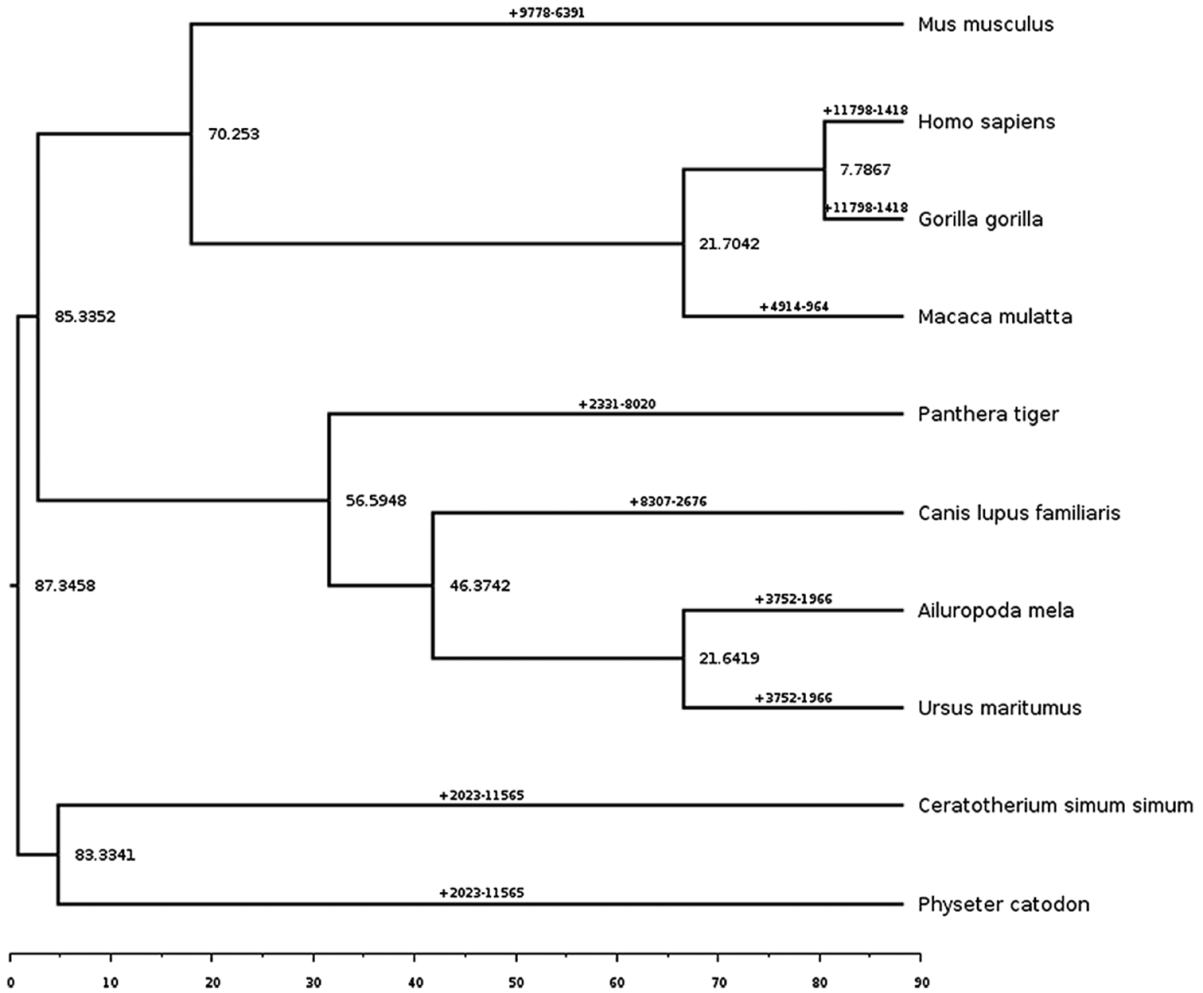
Analysis of the evolution of giant panda gene family. The number of points represent the time of divergence, in millions of years (Myr). The numbers on the branches represent the number of genes, - for contraction, + for expansion.

**Figure 3.**
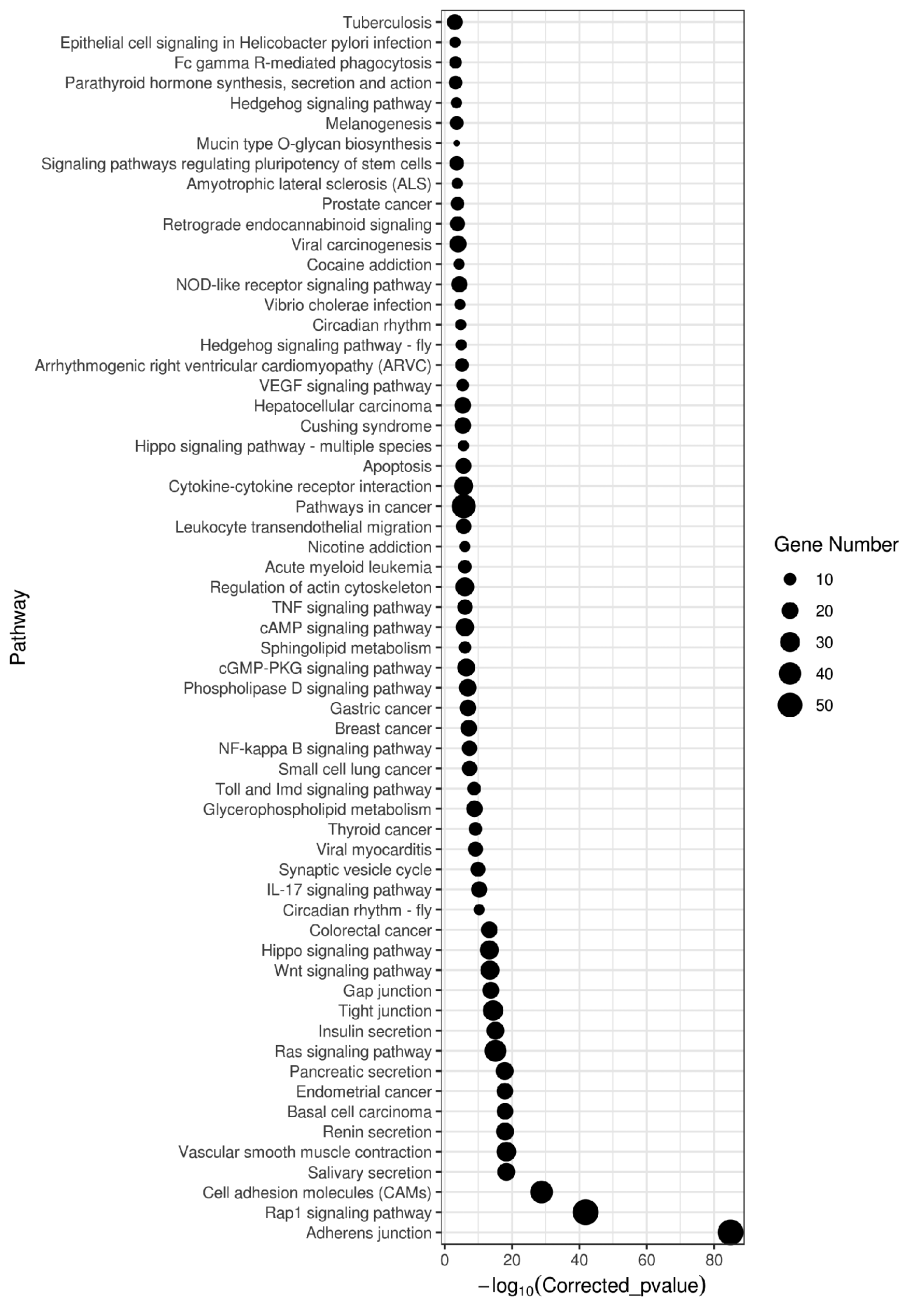
The KEGG enrichment analysis of the giant panda expansion gene family. The abscissa is the pair value of the corrected p value, and the corrected p < 0.05 is taken as the threshold value.

The phylogenetic tree constructed using all 10 mammalian species based on single-copy gene family data estimated the time of divergence between giant panda and polar bear to be 21 Myr (Fig. 2). The giant panda had a 3752-gene family expansion and a 1966-xcgene family contraction (Fig. 2). The KEGG enrichment analysis of the expansion gene families showed an enrichment in digestion-related pathways such as salivary secretion, pancreatic secretion, insulin secretion and parathyroid hormone synthesis, secretion and action (Fig. 3). The KEGG enrichment of the contractile gene was not significant, but the cytochrome CYP450 gene family, which normally shrinks in carnivores, was not found in the giant panda’s contractile gene family.

### Positive selection gene

**Figure 4.**
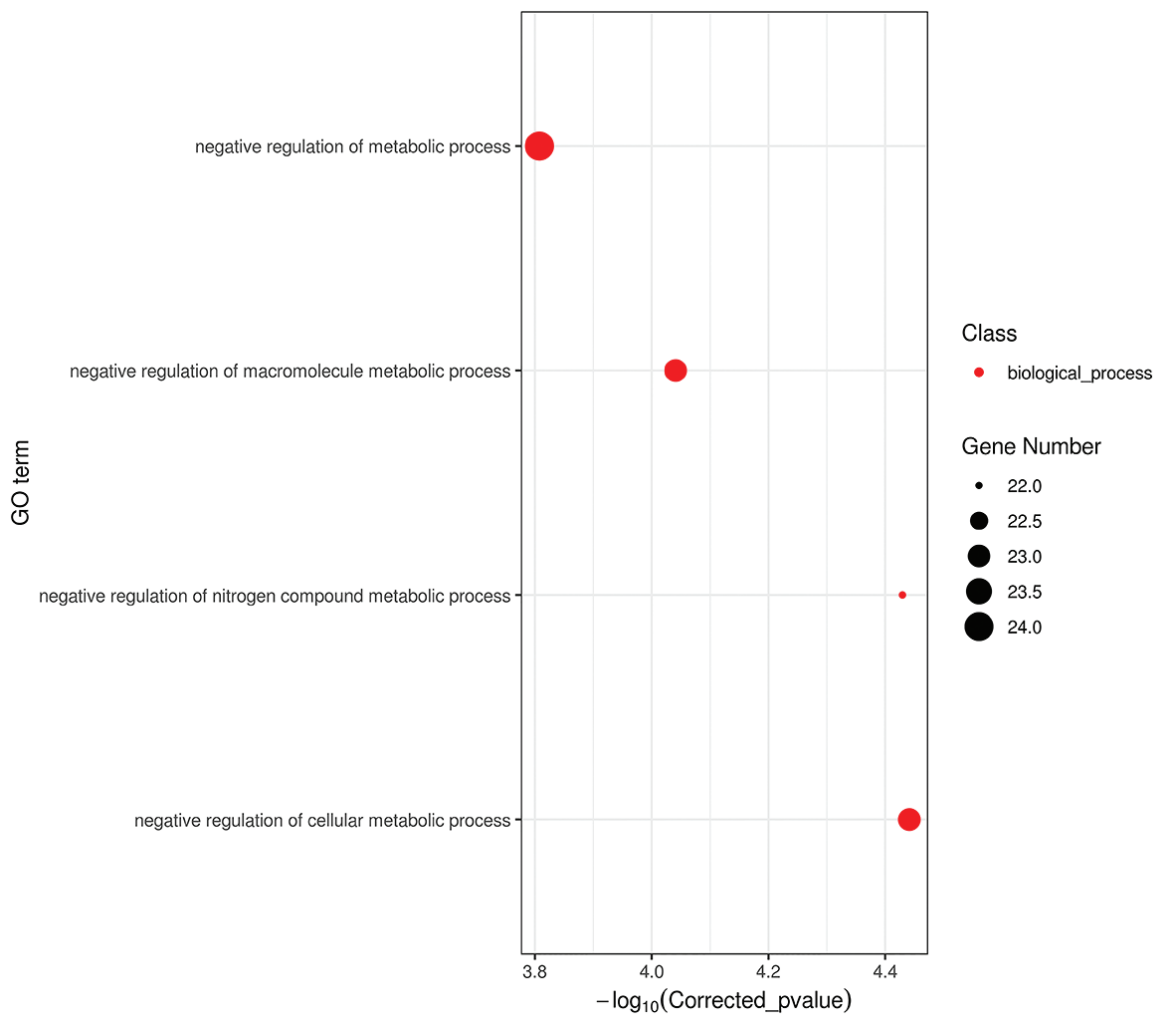
The GO enrichment analysis of giant panda positive selection gene. The abscissa is the pair value of the corrected p value, and the corrected p < 0.05 is taken as the threshold value.

A total of 93 genes were identified to be under positive selection in the giant panda branch. In the GO enrichment analysis, it was found that the positive gene enrichment was selected for the negative regulation of the cellular metabolic process, negative regulation of the nitrogen compound metabolic process, negative regulation of the macromolecule metabolic process and negative regulation of the metabolic process (Fig. 4). The biological function of all genes was analyzed by the KEGG pathway. The following genes were detected in giant pandas: cAMP-responsive element binding protein 3-like 1(CREB3L1), cytochrome P450 family 2, subfamily S, polypeptide 1(CYP450 2S1) and other metabolism-related genes. CREB3L1 regulates glucose metabolism, while CYP450 2S1 is a key gene involved in vitamin A metabolism. These may have played a key role in the giant pandas’ adaptation to the bamboo diet.

## Discussion

The carnivorous diet of the ancestors of the giant panda has gradually evolved into a strict bamboo diet since the late Pliocene or early Pleistocene. In order to adapt to this change in diet, giant pandas developed adaptive evolution genetically, physiologically and morphologically. By comparing the genes that the giant panda shares with several carnivorous and herbivorous species, we found that they share genes with similar smell and perception functions (Charlton et al. 2009). This is consistent with previous findings that giant pandas have an acute sense of smell (Reik and Walter 1998; Zhu et al. 2017). Giant pandas are active at night and during the day, and their keen sense of smell enables them to move around better in dark conditions at night (Reik and Walter 1998). Giant pandas also use olfactory cues to respond to mating signals and to determine the different kinds of food (Zhu et al. 2017). However, in terms of the microbial defense mechanisms (Lecellier et al. 2005), giant pandas tend to act like a herbivore. Giant pandas show a positive regulation of hydrolase activity (Rammler et al. 1973), a negative regulation of biological stimulation, and a positive regulation of catalytic activity (Antonov et al. 1988). These functions might help giant pandas defend against vegetative toxins in bamboo, and at the same time effectively digest and absorb the nutrients in bamboo. Studies have shown that bamboo contains cyanide, but pandas can break cyanide down in their digestive system (Huang et al. 2016).

Giant pandas have several families of amplified genes, among which salivary secretion, pancreatic secretion, insulin secretion and parathyroid hormone synthesis, secretion and function pathways may be important for the digestion and adaptation of giant pandas to bamboo. The sialaden and pancreas are important glands for the digestive system; the increased saliva secretion can help giant pandas lubricate the gut for digesting the starch in bamboo (Maynard and Loosli 1969). The amylase gene copy number of the giant panda is higher than that of the genomes of carnivores that we queried, which is consistent with the fact that the giant panda eats mainly hemicellulose and starchy materials (Zhang et al. 2018). Bamboo is mostly indigestible, and its content of starch and other carbohydrates could be the giant panda’s main source of energy. The pancreas secretes a variety of digestive enzymes which can help giant pandas digest better the macromolecules in bamboo and facilitate the absorption of nutrients (Eckert et al. 1988; Wu et al. 2017). The increase in the salivary and pancreatic secretions may be a key digestive strategy for giant pandas in response to the dietary change to bamboo. Insulin is one of the hormones secreted by the pancreas, which promotes the muscle and liver to absorb, store and utilize glucose; in addition, insulin promotes the synthesis and storage of fat and protein, and inhibits the decomposition and utilization of fat and protein (Schmidt-Nielsen 1997; Hill et al. 2004). Bamboo has few nutrients and is difficult to digest. Insulin secretion could help giant pandas transfer and store digested nutrients with maximum efficiency. Parathyroid hormone mainly increases serum calcium and decreases serum phosphorus. The giant panda may benefit from parathyroid hormone in promoting the renal distal tubule and collecting duct to reabsorb calcium ions, helping the body to reduce calcium loss and increase the use of calcium (Maynard and Loosli 1969; Eckert et al. 1988; Schmidt-Nielsen 1997; Hill et al. 2004). Further studies to determine whether there are changes in the expression of the aforementioned gene products are warranted. These studies will help to verify the adaptability of giant pandas in changing feeding habits from meat to plants.

In the present study, 93 genes were found under positive selection in the genome of the giant panda. The GO enrichment analysis detected that these genes were concentrated in the negative regulation mechanism of metabolism. Activation of this mechanism could reduce their own metabolism and energy needed for adaptation to ecological changes. The results of the present study are consistent with the report showing that in order to cope with the low energy and nutrition content of the bamboo diet, giant pandas reduce their own metabolism, reduce behavioral activities, and eat voraciously to meet their own energy demand (Nie et al. 2015a). At the same time, through the KEGG pathway of the positive selection gene (Zhang et al. 2014; Fuhrmeister et al. 2016), CREB3L1 could suppress the glucose metabolism-related gene expression; and down-regulation of CREB3L1 could weaken the effect of sugar dysplasia and control of glucose levels in the body (Greenwood et al. 2015). This gene is also involved in the pancreatic function, which helps pandas digest bamboo (KEGG reference pathway: ko04152). Bamboo is the sole source of vitamins for pandas. To meet this nutritional challenge, pandas need to be more efficient at absorbing and using nutrients. Pandas can synthesize retinoids from beta-carotene in bamboo, which can be converted to vitamin A, which is metabolized to retinoic acid. Through the study of convergent evolution of giant panda and red panda, it was found that the CYP450 gene family of the giant panda plays an important role in the metabolism of vitamin A (Hu et al. 2017). We also found a CYP450 2S1 gene in our study. CYP450 2S1 is a member of the CYP450 gene family, which is involved in the nutritional metabolism of arachidonic acid and vitamin A in giant pandas (Molotkov et al. 2004). The excessive accumulation of retinoic acid causes harm to physical development (KEGG reference pathway: ko00830).

Other genes hint at adaptations in other directions. For example, the HSD11B2 gene can encode type 2 11β-hydroxysteroid dehydrogenase and participate in intracellular homeostasis, and convert cortisol to cortisone. It is an inactive corticosteroid that can prevent obesity and high blood pressure (Mune et al. 2013). In the present study, we found that LRPAP1, a gene related to myopia, was elevated in giant pandas, which provided a new clue for the giant pandas’ poor eyesight (Aldahmesh et al. 2013). The protein/enzyme expression from these genes need to be investigated in future studies to verify the results of the present study.

## Conclusion

Changes in diet composition during animal evolution likely created strong selective pressures in multiple biological processes. The giant pandas’ dietary habits have shifted dramatically from a carnivorous diet to a strict bamboo diet. The reasons for the changes in the diet of giant pandas are still largely unknown. With the development of high throughput, next generation sequencing technology and the open availability of genome-wide data, we explored the adaptability of giant pandas from a genomic perspective.

In this study, the comparative genomics of species with different feeding habits was used to elucidate the genes responsible for the adaptation of giant pandas to herbivory. The giant panda retains the characteristics of a carnivore in its sensory abilities, but is similar to a herbivore in terms of physiology, such as digestion. These adaptations also help the panda digest bamboo and metabolize nutrients by regulating its digestive and endocrine secretions. Here, we elucidated the molecular/genomic mechanism of giant pandas’ adaptation to dietary changes and the adoption of dietary specializations in the digestive system. However, the function of these genes in giant pandas need to be further verified. In response to changes in diet, the giant panda’s genome has undergone important evolutionary adaptations that help it better digest bamboo and metabolize nutrients.

This study also provides new perspectives and insights for the adaptive evolution of giant pandas. In addition, these findings also provide the molecular theoretical basis for the adaptation mechanism of giant panda’s dietary changes, and present additional information that can be used for the management and protection of this endangered species.
